# Impact of maternally derived pertussis antibody titers on infant whole-cell pertussis vaccine response in a low income setting

**DOI:** 10.1016/j.vaccine.2018.09.045

**Published:** 2018-11-12

**Authors:** Romesa Ibrahim, S. Asad Ali, A. Momin Kazi, Arjumand Rizvi, L. Beryl Guterman, Robert A. Bednarczyk, Ellie Kim, SoHee Park, Simon Paulos, Amilia Jeyachandran, Divya Patel, Yamini Gorantla, Emily Wong, Gowrisankar Rajam, Jarad Schiffer, Saad B. Omer

**Affiliations:** a Department of Paediatrics and Child Health, Aga Khan University, Karachi, Pakistan; b Department of Global Health, Emory University, Atlanta, Georgia; c Department of Epidemiology, Emory University, Atlanta, Georgia; d Department of Pediatrics, Emory University, Atlanta, Georgia; e Department of Emory Vaccine Center, Emory University, Atlanta, Georgia; f MPIR Laboratory, Division of Bacterial Diseases, Centers for Disease Control and Prevention, Atlanta, Georgia

**Keywords:** Pertussis vaccine, Maternal vaccination, Immunogenicity, Immune blunting, DTwP

## Abstract

**Background:**

Maternal vaccines against pertussis are not yet recommended in the developing world. Besides unclear burden estimates, another concern is that transplacental transfer of maternal pertussis antibodies could result in attenuation of the immune response to whole cell pertussis (DTwP) primary vaccination series in infants. This study was taken up to determine whether higher levels of maternal pertussis antibodies attenuate immune response of infants to DTwP vaccination series given at 6–10–14 weeks of age.

**Methodology:**

A total of 261 pregnant women and their infants from four low-income settlements in Karachi, Pakistan were enrolled in this study. The study endpoints were infant antibody titers for Pertussis toxin (PTx), Filamentous hemagglutinin antigen (FHA), Pertactin (PRN) and Fimbriae type 2/3 (FIM) – from birth through 18 weeks of age. Cord blood or pre-vaccine pertussis antibody titers indicate the concentration of maternal antibodies transferred to infants. Linear regression models were used to determine the association between higher maternal antibody titers and infant immune response to DTwP vaccine. Geometric Mean Ratio (GMR) was calculated as the ratio of infant antibody titers at specified time points against the maternal antibody titers at the time of delivery.

**Results:**

At eighteen weeks of age, the adjusted β regression coefficient for PTx was 0.06 (95% CI: -0.49-0.61), FHA 0.02 (95% CI: -0.26 -0.29), PRN 0.02 (95%CI -0.38- 0.43), and FIM 0.17 (95%CI: -0.21-0.54). Among infants who received at least two doses of DTwP vaccine, higher maternal antibody titers did not have any attenuating effect on infant post-immunization antibody titers against all four pertussis antigens.

**Conclusion:**

Maternal pertussis antibodies did not attenuate infant’s immune response to pertussis antigens in DTwP primary vaccine given at 6–10–14 weeks of age.

## 1 Introduction

Most childhood primary Diphtheria, Tetanus and whole cell Pertussis (DTwP) vaccination series in low-and-middle-income countries (LMICs) follow the 6, 10, and 14 weeks immunization schedule [1]. Infants too young to be completely vaccinated are not adequately protected until they are several months old and therefore are more susceptible to acquiring infections, such as pertussis [2]. Maternal vaccination against pertussis during pregnancy can address this immunity gap in young infants through transplacental transfer of maternal pertussis antibodies. In addition, prevention of pertussis in the mother may potentially offer protection to the infant from acquiring pertussis in early infancy. Even though pertussis vaccine as Tdap is recommended for pregnant women in both the UK and the US, it is not part of routine antenatal care in developing countries where the burden of pertussis is likely to be much higher [5], [6].

There is a concern that the transplacental transfer of maternal pertussis antibodies to the infant could result in attenuation of immune response to the primary childhood pertussis vaccination series, also known as “blunting”. Several studies have examined the risk of blunting among infants receiving the acellular pertussis vaccine. However these results have not been conclusive [7], [8].

There are fewer studies assessing the risk of blunting among infants that receive the whole cell pertussis vaccine [4], [9]. A study conducted by Englund et al. showed attenuation of immune response to whole-cell pertussis vaccine administered among infants (2–4–6 months) born to mothers with high pertussis maternal antibody titers [4].

However, no studies to date have assessed the impact of maternal antibody titers on pertussis response in DTwP vaccination series administered at 6–10–14 weeks, the schedule used most commonly in LMICs, and covering a majority of the world’s birth cohort. In this study, we assess the impact of pertussis maternal antibodies (acquired from childhood pertussis immunization or pertussis infection) on the DTwP immune response in infants that received primary vaccination series given at 6–10–14 weeks of age.

## 2 Methodology

### 2.1 Surveillance

The Prevention of Pertussis in Young Infants in Pakistan (PrePY) Baseline Surveillance study was conducted from February 2015 to August 2016 in four low-income settlements of Karachi, Rehri Goth, Ibrahim Hyderi, Bhains colony, and Ali Akbar Shah. The Department of Pediatrics and Child Health of the Aga Khan University Hospital (AKUH) has been operating primary healthcare centers in these areas with active demographic surveillance system for several years. We registered 261 healthy, pregnant women randomly on or after 27 weeks of gestation or mothers who gave birth within 72 h prior to registration. Few subjects were lost to follow-up to reasons such as refusal for blood draw after delivery, molar pregnancy, still birth, death after delivery and refusal to continue in the study (Fig. S3). Baseline maternal characteristics (age, education, antenatal care, TT vaccination, body mass index and hypertensive status) were collected at the time of enrollment. Since it is not recommended in LMICs, mothers were not vaccinated with TDaP during pregnancy. Infants born to these mothers were followed from birth through eighteen weeks of age. Baseline infant characteristics (gender, gestational age, weight, and head circumference) were collected at the time of delivery. Infant DTwP vaccination status was obtained at the 6th, 10th and 14th weeks after delivery. The surveillance methods have been previously described in detail [2].

### 2.2 Blood specimen collection

One blood draw (∼7 mL) was made from the enrolled mothers within 72 h of delivery. Three blood specimens were collected from the infants. The first blood specimen was collected at birth (cord blood) or within 72 h of delivery via a venipuncture. The second blood specimen from infants was collected at 6 weeks of age (prior to receipt of first dose of DTwP) and the third blood specimen was collected at 18 weeks of age. Approximately 3 mL of blood from infants was collected each time. Blood specimens were collected by a trained phlebotomist.

After collection, blood samples were transported to the Infectious Disease Research Laboratory at the AKUH under cold chain maintenance. Sera were separated at the laboratory and stored in cryovials at −80 °C. The sera were transported under appropriate cold-chain management, to the Microbial Pathogenesis and Immune Response (MPIR) Laboratory, Centers for Disease Control and Prevention (CDC), Atlanta USA for testing.

### 2.3 Laboratory procedures

Serum samples were analyzed for antibodies against PTx, PRN, Fim2/3 and FHA using a microsphere based multiplex antibody capture assay (MMACA). Each assay plate included a pertussis human standard reference serum, WHO 06/140, diluted 4-fold for 8 dilutions starting at 1/20; and an internal quality control (QC) serum, WHO 06/142, diluted 2-fold for 4 dilutions starting at 1/400. Test serum samples were diluted 2-fold for 7 dilutions starting at 1/50. IgG-free human serum (Sigma, St. Louis, MO, USA), and assay buffer blanks were included in each assay plate as reagent controls. Replicates were maintained for standard, QC serum and blanks. All QC, reference standard and sample dilutions were carried out in a 96-well round bottom titer plate (CLS3799, Sigma, St. Louis, MO). A 96-well multiscreen HTS filter plate (MABVN1250, Millipore Corp, Billerica, MA) pre-wet with 100 μL assay buffer (0.1% BSA (wt/vol) in PBS) was aspirated and 25 μL/well of pertussis antigen-conjugated microspheres (multi-plex; 2500 microspheres/region/well) were transferred. From the dilution plate, 25 μL reference standard, QCs and serum samples were transferred to the filter plate with microspheres and incubated in dark for 40 ± 10 min at RT with 150 RPM agitation in a horizontal orbital agitator. The plate was aspirated and washed 3 times with 100 μL assay buffer. To each well, 50 μL of a 1/200 dilution of R-PE conjugated Goat anti-human Fcγ specific IgG (GTIGF-001, Moss Inc. Pasadena, MD) in PBS was added and incubated in dark for 20 ± 10 min at RT with 150 RPM agitation in a horizontal orbital agitator. The plate was aspirated, washed 3 times with 100 μL assay buffer and the microspheres were resuspended in 130 μL assay buffer. The plate was read in a Luminex 200 reader (Luminex Corp, Houston, TX) and the data analyzed for the antibody concentration using a SAS version 9.3 (SAS Institute Inc, Cary, NC) running a MMACA customized endpoint calculation algorithm. The reportable value (RV) of the assay is the mean of two independent tests and expressed as the serum concentration of anti-pertussis antigens specific IgG in IU/mL.

### 2.4 Statistical analysis

The primary outcomes were antibody titers for four pertussis antigens (PTx, FHA, PRN and FIM) in cord blood and infant serum at birth, at six weeks, and at eighteen weeks. Geometric mean concentration (GMC) with 95% confidence interval was computed, stratified by DTwP vaccine dose, for all pertussis antigens at specified time points. Geometric Mean Ratios (GMR) with 95%CI were calculated to compare the antibody titer of infants at each time point (birth, 6 weeks and 18 weeks) to the maternal antibody titer at delivery. Half-lives of all pertussis antibodies were determined in unvaccinated control infants.

Crude and multivariable linear regression models were used to determine the impact of higher maternal antibody titers, at the time of delivery, on infant vaccine response at specified time points. The multivariable model adjusts for birth weight, gestational age (determined from date of last menstrual period as reported by pregnant mothers) and gender. All infants who received at least 2 doses of DTwP vaccine were included in this analysis. Both maternal and infant antibody concentrations were log2-transformed and maternal antibody titers were included in the model as a continuous variable. The regression coefficient, β, from this model and their 95%CIs were presented as the measure of association between infant antibody titers at the specified time point (birth, 6 weeks and 18 weeks) to maternal antibody titers at delivery. Where negative β regression coefficients imply that infant antibody titers decline with increasing maternal antibody levels at delivery, positive numbers imply that infant antibody titers increase with increasing maternal antibody levels at delivery and null values (β=0) imply no association between infant and maternal antibody titers at delivery. For comparison, sensitivity analysis was conducted using dichotomized (above and below median) maternal antibody titers keeping below median as the reference category.

Approval from Ethical Review Committee/Institutional Review Boards of the AKU, Emory University, and Centers for Disease Control Prevention as well as written informed consent from the study subjects, was obtained before commencement of this study.

## 3 Results

A total of 261 healthy, pregnant women in their third trimester of pregnancy met the eligibility criteria and were included in the current study. Serum samples for serologic analysis were collected from 219/261 (83.9%) pregnant women within 72 h of delivery. Table 1 summarizes the baseline maternal characteristics of the pregnant women in our study cohort. Maternal age was 26 years, on average with almost two-thirds of mothers having no formal education. Approximately 3% of the mothers were underweight (BMI < 18.5) and only 2% were hypertensive at the time of enrollment.

**Table1:** Baseline maternal characteristics of pregnant women in the study.

Maternal characteristics	Mean ± SD or N (%)
Maternal age	
16–20 years	50 (19.2)
21–25 years	82 (31.4)
26–30 years	75 (28.7)
>30 years	54 (20.7)
Mean ± SD	26.4 ± 5.8
n	261

Maternal education	
No formal education	174 (66.7)
Formal education received	87 (33.3)
n	261

ANC	
None	118 (45.2)
1	13 (5)
2	21 (8.1)
3	26 (10)
>3	83 (31.8)
n	261

ANC skilled provider	
Any skilled provider	93 (85.3)
Others	16 (14.7)
n	109

TT vaccines doses	
None	94 (36)
1	84 (32.2)
2	81 (31)
3	2 (0.8)
N	261

Body Mass Index*	
Underweight	7 (3.8)
Normal	75 (40.5)
Overweight/Obese	103 (55.7)
n	185

Hypertension	
Yes	4 (2.1)
No	191 (98)
n	195

Note: Maternal education was assessed in terms of the number of completed years of schooling, where if a woman ever attended school she was categorized as received a formal education and if she did not receive any years of schools she was categorized as No Formal Education.

Abbreviations: ANC = Antenatal care, TT = Tetanus Toxoid.

* BMI cutoffs were defined as follows: Underweight (BMI < 18.5), Normal (BMI 18.5-25), Overweight (BMI > 25).

In this study, a total of 222 infants were followed from birth until the end of study follow-up (i.e. 18 weeks of age). Serum samples for serologic analysis were collected from 206/222 (93%) infants at birth (cord/serum samples), 133/222(60%) after six weeks and 143/222 (64%) infants after 18 weeks. Table 2 summarizes the baseline characteristics of the infants followed until the end of study period. In our study cohort, the mean (SD) gestational age at birth was 34.6 (4.4) weeks. Almost half of all infants were boys and the mean (SD) birth weight in kilograms (kg) of infants in our study cohort was 2.8 (0.4). There was no significant difference in infant antibody titers between infants whose first blood specimen was collected at birth (cord blood) and infants whose first blood specimen was collected within 72 h of delivery via a venipuncture (p = 0.259, Table S9).

**Table 2:** Baseline characteristics and vaccination status of infants until the end of study follow-up (18 weeks).

Infant characteristics	Mean ± SD or N (%)
Gender	
Male	121 (54.3)
Female	102 (45.7)
n	223

Age at first blood draw (days)	
Mean ± SD	2.2 ± 3.4
n	200

Weight (Kg)	
Mean ± SD	2.8 ± 0.4
n	218

Length (cm)	
Mean ± SD	47.9 ± 3.7
n	204

Head circumference (cm)	
Mean ± SD	33.2 ± 1.7
n	205

Vaccination status*	
DTwP 1 dose	163 (73.2)
DTwP 2 doses	101 (45.1)
DTwP 3 doses	28 (12.5)
DTwP (at least one dose)	163 (73.2)
n	215

* Infants were vaccinated according to the EPI 6–10–14 week immunization schedule.

Approximately 70% of the infants received at least one dose of DTwP vaccine whereas 45% and 12.5% of the infants were reported to have received two doses and all three doses of DTwP vaccine respectively, at 6–10–14 weeks of age. There were no significant differences in demographic characteristics between infants who received at least one, two or three doses of DTwP (Tables S7, S8). There was some variation in the age at which infants received each dose of DTwP, however overall vaccine doses were given within recommended age range (Table S10).

Geometric mean ratios indicate that maternal antibody titers at delivery did not blunt infant vaccine response, among infants who received at least two doses of DTwP vaccine, for all pertussis antigens (PTx, FHA, PRN and FIM, Table 3). In the adjusted model (adjusting for gestational age at birth, gender and birth weight), we observed that infant antibody titers increase significantly with increasing maternal antibody levels at delivery against all pertussis antigens (PTx, FHA, PRN and FIM) at birth and at six weeks (Table 4). Breastfeeding status was not included in the multivariable model because it had no meaningful effect on the outcomes of interest (Tables S5, S6). Also, limiting to infants who received at least two doses of DTwP vaccine, higher maternal antibody titers did not have a significant effect on infant post-immunization (18 weeks) antibody titers, against all pertussis antigens (PTx, FHA, PRN and FIM) in the adjusted model. These results were confirmed when maternal antibody titers were modeled as a binary variable (above or below median- keeping below median as the reference category, Table S2).

**Table 3:** GMR with 95% CI-for children who received no doses and for children who received at least two doses of DTwP vaccine, per pertussis antigen at birth and at 6 and 18 weeks of age.

Geometric Mean Ratio (GMR)	Unvaccinated			Vaccinated		
	At Birth	At 6 weeks	At 18 weeks	At Birth	At 6 weeks	At 18 weeks
PTx						
GMR (95%CI)	0.77 (0.62, 0.96)	0.36 (0.26, 0.49)	0.21 (0.07, 0.6)	0.83 (0.69, 1.01)	0.32 (0.26, 0.39)	1.9 (1.01, 3.58)

FHA						
GMR (95%CI)	1.01 (0.82, 1.25)	0.42 (0.32, 0.56)	0.15 (0.08, 0.28)	1.07 (0.93, 1.24)	0.48 (0.4, 0.59)	0.62 (0.44, 0.89)

PRN						
GMR (95%CI)	0.54 (0.42, 0.69)	0.38 (0.25, 0.55)	0.47 (0.19, 1.17)	0.45 (0.36, 0.57)	0.25 (0.19, 0.32)	2.42 (1.51, 3.89)

FIM						
GMR (95%CI)	0.78 (0.62, 0.99)	0.51 (0.3, 0.87)	2.96 (0.79, 11.12)	0.91 (0.69, 1.19)	0.49 (0.38, 0.64)	5.08 (2.76, 9.36)

**Table 4:** Association between maternal antibody titers (at the time of delivery) and infant antibody titers as measured by linear regression coefficient, β, with 95% CIs for children who received at least two doses (n = 129) of DTwP vaccine, per pertussis antigen at various time points in both unadjusted and adjusted (adjusted for birth weight, gestational age and gender) linear regression models.

Regression coefficients (α,β) with 95% confidence interval & P-values***	Unadjusted model			Adjusted model		
	At Birth	At 6 weeks	At 18 weeks	At Birth	At 6 weeks	At 18 weeks
PTx						
α (95% CI)*	0.12 (−0.32, 0.57)	−1.43 (−1.96, −0.9)	2.75 (1.38, 4.13)	2.32 (−0.62, 5.26)	0.46 (−2.95, 3.87)	10.3 (1.99, 18.6)
p-value	0.5834	<0.0001	0.0001	0.1204	0.7893	0.0161
β (95%CI)**	0.83 (0.67, 0.99)	0.9 (0.71, 1.09)	0.14 (−0.37, 0.64)	0.79 (0.61, 0.98)	0.81 (0.58, 1.05)	0.06 (−0.49, 0.61)
p-value	<0.0001	<0.0001	0.5863	<0.0001	<0.0001	0.8293

FHA						
α (95% CI)*	0.68 (−0.32, 0.57)	−0.33 (−1.96, −0.9)	2.86 (1.38, 4.13)	3.52 (1.5, 5.55)	2.46 (−0.72, 5.64)	3.84 (−0.18, 7.87)
p-value	0.0172	0.4064	<0.0001	0.0009	0.1272	0.0608
β (95%CI)**	0.86 (0.74, 0.99)	0.83 (0.66, 1)	0.14 (−0.1, 0.37)	0.75 (0.61, 0.88)	0.73 (0.52, 0.94)	0.02 (−0.26, 0.29)
p-value	<0.0001	<0.0001	0.2512	<0.0001	<0.0001	0.9084

PRN						
α (95% CI)*	−1.22 (−1.79, −0.66)	−1.61 (−2.2, −1.02)	3.08 (2.07, 4.08)	0.97 (−2.59, 4.54)	0.23 (−3.76, 4.22)	4.04 (−2.09, 10.16)
p-value	<0.0001	<0.0001	0.1757	0.5881	0.9086	0.1919
β (95%CI)**	1.03 (0.84, 1.23)	0.83 (0.63, 1.03)	0.23 (−0.11, 0.57)	0.91 (0.68, 1.14)	0.79 (0.53, 1.05)	0.02 (−0.38, 0.43)
p-value	<0.0001	<0.0001	0.1757	<0.0001	<0.0001	0.9166

FIM						
α (95% CI)*	0.12 (−0.4, 0.64)	−0.45 (−0.93, 0.03)	4.23 (3.28, 5.19)	1.77 (−2.71, 6.25)	0.53 (−3.38, 4.44)	5.91 (−1.78, 13.59)
p-value	0.6446	0.0659	<0.0001	0.4322	0.7871	0.1293
β (95%CI)**	0.88 (0.72, 1.04)	0.74 (0.59, 0.89)	0.04 (−0.27, 0.35)	0.88 (0.67, 1.09)	0.66 (0.48, 0.85)	0.17 (−0.21, 0.54)
p-value	<0.0001	<0.0001	0.1757	<0.0001	<0.0001	0.9166

Note: Linear regression models using the log2 transformed antibody titers were used to determine the impact of maternal titers at the time of delivery on infant vaccine response at specified time points.*α is the regression intercept. ** Negative β coefficients imply that infant antibody titers decline with increasing maternal antibody levels at delivery, positive numbers imply that infant antibody titers increase with increasing maternal antibody levels at delivery and a β coefficient of zero indicates no association between infant antibody titers and maternal antibody levels at delivery. ***P-value < 0.05 was considered significant. Abbreviations: FHA = Filamentous hemagglutinin, FIM = Fimbriae type 2/3, PRN = Pertactin, and PTx = Pertussis toxin.

**Fig. 1:**
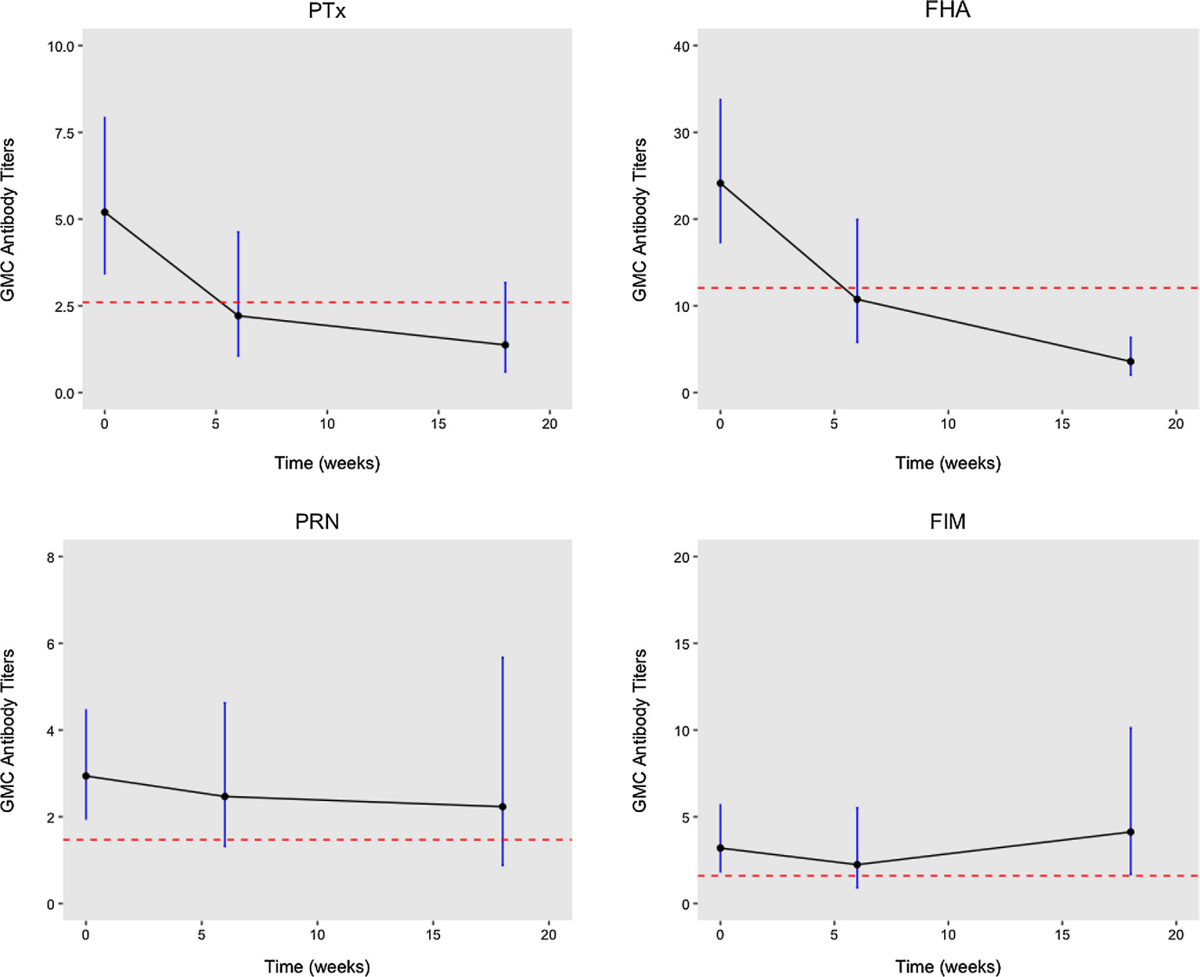
Geometric mean concentrations (GMC) with 95% confidence interval (CI) of infant pertussis antibody titers and their half-lives for infants who did not receive any vaccination. Abbreviations: FHA = Filamentous hemagglutinin, FIM = Fimbriae type 2/3, PRN = Pertactin, and PTx = Pertussis toxin. Note: I bars indicate 95% CIs. (--- one half infant antibody titer at birth). Outliers (defined as greater than two standard deviations from the mean, n = 1) were excluded (See Fig. S2 for GMC with 95% confidence intervals with outliers included). Birth = Infant cord blood/sera (n = 55); 6 (n = 18) and 18 weeks (n = 19) = Infant sera.

Table S1 lists the geometric mean concentrations (GMC) of all pertussis antigens (PTx, FHA, PRN and FIM), according to time of sampling and infant vaccination status. Our results demonstrated significantly higher GMCs for all pertussis antigens (except FIM) at 18 weeks in those who received at least two doses of DTwP vaccine as per WHO recommended 6–10–14 week EPI vaccination schedule used in LMICs- compared to unvaccinated (Fig. S1). We found that in unvaccinated infants, the half-life of antibodies against PTx and FHA pertussis antigens was approximately six weeks (Fig. 1). However, given the data, the half-life of antibodies to FIM and PRN antigens were not clear (Fig. 1).

## 4 Discussion

We assessed whether higher maternal pertussis antibody titers attenuate vaccine response to childhood DTwP primary vaccination series (6–10–14 week immunization schedule) in young infants in a low income setting. We found no association between higher maternal pertussis antibody titers and post-immunization (18 weeks) pertussis antibody titers in infants who had received at least two doses of DTwP vaccine. Further, this study demonstrated that higher maternal antibody titers significantly increased infant antibody titers at birth and at six weeks. While infant antibody titers decreased at 18 weeks, this change was small and not significant. We observed that the estimated duration of half-life of the maternally transferred antibody against the pertussis antigens PTx and FHA were approximately six weeks. Likewise, a study conducted by Van Savage et al. demonstrated that the duration of half-life of maternally transferred antibody against PTx and FHA pertussis antigens was around six weeks [3].

Previous studies have reported varying associations between higher maternal antibody titers and attenuation of infant vaccine response. A study conducted by Englund et al. [4], found higher levels of pre-existing maternal antibody titers against PTx were associated with substantial reductions in the post-immunization titers among infants who had received three doses of DTwP (2–4–6 months immunization schedule) [4]. In contrast, our findings demonstrate that the infant (PTx) response to DTwP vaccine was not adversely affected in the presence of higher levels of maternal antibody titers. One major difference is Englund et al. assessed infant immune responses among those who received 3 doses of DTwP vaccine while our study assessed infant immune response among those who received at least 2 doses of the DTwP vaccine. In addition, a meta-analysis conducted by Voysey et al. [8] found significant blunting of infant vaccine response to DTaP-specifically against PTx, FHA and PRN pertussis antigens- among infants born to mothers who received acellular pertussis vaccination during pregnancy. Despite the differences in DTwP primary immunization schedules (6–10–14 weeks versus 2–4–6 months), both our study and the study conducted by Englund et al. [4], support the introduction of comprehensive pre-natal immunization programs which could potentially result in the closure of the susceptibility gap for pertussis in young infants.

Our results are consistent with those obtained from a randomized controlled trial conducted in Vietnam [10], which demonstrated no blunting of immune response against PTx and FHA pertussis antigens in infants who received DTaP vaccine (2–3–4 months primary immunization schedule) in the presence of higher maternal antibody titers obtained from mothers who were vaccinated with DTaP during pregnancy. However, the type of pertussis vaccine used (acellular pertussis vaccine versus whole-cell pertussis vaccine) and the dosing schedule (2–3–4 months versus 6–10–14 weeks) were different between these two studies.

There are several strengths in our study which merit mention. First, we followed a large cohort of infants closely over an eighteen week period of follow-up, which was a challenging task in a developing country setting. Second, we included potential confounding factors such as gestational age, gender and birth weight in our adjusted model. Finally, the present study is the first to describe the impact of high maternal antibody titers on infant immune response to DTwP vaccine (6–10–14 week immunization schedule), used in LMICs.

We were limited by the lack of maternal history regarding parity, childhood DTwP vaccination status and exposure to pertussis disease. Few subjects were lost to follow-up due to reasons such as refusal for blood draw after delivery, molar pregnancy, still birth, death after delivery and refusal to continue in the study. Despite this, subject retention and follow- up in our study was sufficient to provide required statistical strength to draw comprehensive conclusions.

In summary, we found no blunting association between higher pertussis maternal antibody titers and infant immune response to childhood DTwP vaccine given at 6–10–14 weeks of age in LMICs.

## Financial support

This study was supported by a grant from the Bill & Melinda Gates Foundation (OPP1092014)

## Potential conflicts of interest

All authors: No reported conflicts.

## Author summary

Maternal vaccines against pertussis are not yet recommended in the developing world due to unclear burden estimates and possible infant vaccine response attenuation by maternal antibodies. Our findings suggest that maternal pertussis antibodies do not attenuate serologic response in infants to pertussis (DTwP) primary vaccination series.
