# Supplementary material for: Impact of maternally derived pertussis antibody titers on infant whole-cell pertussis vaccine response in a low income setting
Source: Vaccine. 2018 Nov 12;36(46):7048–53. doi: 10.1016/j.vaccine.2018.09.045 (PMC6219892; doi:10.1016/j.vaccine.2018.09.045)
Supplement: Supplementary data 1 [file mmc1.docx]

**Supplemental Materials:**

Contents

[**Table S1**. Comparison of Geometric mean concentrations (GMC) of maternal and infant antibody titers-per pertussis antigen at various time points- in those who were unvaccinated compared to those who received at least two doses of DTwP vaccine.	3](#_Toc525651813)

[**Table S2.** Influence of maternal antibody titers (at the time of delivery) - which were dichotomized as above or below median (relative to below median category of maternal antibody titers)-on infant vaccine response, for children who received at least two doses (n=129) of DTwP vaccine, per pertussis antigen at various time points in both unadjusted and adjusted (for birth weight, gestational age and gender) models.	4](#_Toc525651814)

[**Table S3**. Association between maternal antibody titers (at the time of delivery) and infant antibody titers as measured by linear regression coefficient, β, with 95% CIs - for children who received three doses of DTwP vaccine (n=28), per pertussis antigen at various time points in both unadjusted and adjusted (for birth weight, gestational age and gender) models.	6](#_Toc525651815)

[**Table S5.** Association between maternal antibody titers (at the time of delivery) and infant antibody titers as measured by linear regression coefficient, β, with 95% CIs for children who received at least two doses (n=129) of DTwP vaccine, per pertussis antigen at various time points in both unadjusted and adjusted (adjusted for birth weight, gestational age, gender and breast feeding status) linear regression models.	10](#_Toc525651816)

[**Table S6.** Influence of maternal antibody titers- which were dichotomized as above or below median (relative to below median category of maternal antibody titers) - on infant vaccine response, for children who received at least two doses (n=129) of DTwP vaccine, per pertussis antigen at various time points in both unadjusted and adjusted (for birth weight, gestational age, gender and breast feeding status) models.	12](#_Toc525651817)

[**Table S7.** Comparison of maternal demographics among those who received 1 vs 2 vs 3 doses of DTwP.	13](#_Toc525651818)

[**Table S8.** Comparison of infant demographics among those who received 1 vs 2 vs 3 doses of DTwP.	15](#_Toc525651819)

[**Table S9.** Comparison between Ab titers in cord blood vs those obtained within 72 hours of delivery.	17](#_Toc525651820)

[**Table S10.** Variability in timing of DTwP doses	17](#_Toc525651821)

[**Fig S1**. Maternal and infant pertussis antibody titers in those who were unvaccinated compared to those who received at least two doses of DTwP vaccine (n=129). Pregnancy = Maternal sera; Birth = Infant cord blood/sera; 6 and 18 weeks = Infant sera	18](#_Toc525651822)

[**Fig S2**. Geometric mean concentrations (GMC) with 95% confidence interval (CI) of infant pertussis antibody titers and their half-lives for infants who did not receive any vaccination. *Abbreviations*: *FHA= Filamentous hemagglutinin, FIM= Fimbriae type 2/3, PRN= Pertactin, and PTx= Pertussis toxin*. *Note: I bars indicate 95% CIs.*(------- one half infant antibody titer at birth). Outliers (defined as greater than two standard deviations from the mean) were included (See Figure 2 for GMC with 95% confidence intervals with outliers excluded).	20](#_Toc525651823)

[**Fig S4.** Comparison of Geometric mean concentrations (GMC) of infant antibody titers who received at least two doses of DTwP vaccine-per pertussis antigen at various time points- in those with high maternal antibody titers at delivery (right) compared to those with low maternal antibody titers at delivery (left). Maternal antibody titers at delivery were dichotomized as above or below median.	21](#_Toc525651824)

# **Table S1**. Comparison of Geometric mean concentrations (GMC) of maternal and infant antibody titers-per pertussis antigen at various time points- in those who were unvaccinated compared to those who received at least two doses of DTwP vaccine.

|  | **PTx** | | **FHA** | | **PRN** | | **FIM** | |
| --- | --- | --- | --- | --- | --- | --- | --- | --- |
|  | **Unvaccinated***  GMC(95%CI) | **Vaccinated****  GMC(95%CI) | **Unvaccinated***  GMC(95%CI) | **Vaccinated****  GMC(95%CI) | **Unvaccinated***  GMC(95%CI) | V**accinated****  GMC(95%CI) | **Unvaccinated***  GMC(95%CI) | V**accinated****  GMC(95%CI) |
| **Maternal antibody titer** | 6.41(4.60-8.93) | 4.77(3.76-6.06) | 23.05(17.40-30.54) | 18.56(14.7423.37) | 6.04(4.39-8.32) | 5.34(4.18-6.83) | 3.98(2.38-6.65) | 4.38(3.1-46.12) |
| **Infant titer at Birth** | 5.20(3.40-7.95) | 4.09(3.09-5.40) | 24.13(17.19-33.89) | 20.57(16.10-26.27) | 2.94(1.93-4.48) | 2.44(1.75-3.41) | 3.20(1.78-5.74) | 4.13(2.77-6.16) |
| **Infant titer at 6 weeks** | 2.30(1.14-4.63) | 1.50(1.09-2.05) | 11.02(6.14-19.78) | 9.28(6.96-12.39) | 2.44(1.35-4.42) | 1.29(0.92-1.80) | 1.94(0.79-4.78) | 2.22(1.53-3.21) |
| **Infant titer at 18 weeks** | 1.57(0.68-3.62) | 8.16(4.53-14.70) | 3.66(2.13-6.29) | 10.74(8.18-14.09) | 2.44(0.99-6.01) | 12.39(8.12-18.90) | 5.48(1.94-15.46) | 19.46(11.79-32.12) |

**Unvaccinated: infants who received no dose of DTwP vaccine; Maternal titer (n) = 61, infant titer at birth (n) = 55, infant titer at six weeks (n) =19, infant titer at eighteen weeks (n) = 20.*

***Vaccinated: infants who received at least two doses of DTwP vaccine; Maternal titer (n) = 99, infant titer at birth (n) = 93, infant titer at six weeks (n) = 79, infant titer at eighteen weeks (n) = 84. Abbreviations: FHA= Filamentous hemagglutinin, FIM= Fimbriae type 2/3, PRN= Pertactin, and PTx= Pertussis toxin. Note: Maternal antibody titers were collected at the time of delivery or within 72 hours of delivery and had the following ranges in IU/mL (min, max): FHA (0.83-367.17), FIM (0.14-651.44), PRN (0.17, 163.67), and PTx (0.35, 120.45). Infant titers at birth were collected from cord-serum samples (n=130) or peripheral blood via venipuncture (n=72) within 72 hours of delivery.*

# **Table S2.** Influence of maternal antibody titers (at the time of delivery) - which were dichotomized as above or below median (relative to below median category of maternal antibody titers)-on infant vaccine response, for children who received at least two doses (n=129) of DTwP vaccine, per pertussis antigen at various time points in both unadjusted and adjusted (for birth weight, gestational age and gender) models.

| ***Regression Coefficients (α,β) with 95% confidence interval & P-values****** | **Unadjusted Model** | | | **Adjusted Model** | | |
| --- | --- | --- | --- | --- | --- | --- |
|  | **At Birth** | **At 6 weeks** | **At 18 weeks** | **At Birth** | **At 6 weeks** | **At 18 weeks** |
| **PTx** | | | | | | |
| **α (95% CI)*** | 0.98(0.54, 1.42) | 0.98(0.54, 1.42) | 3.03(1.94, 4.13) | 3.87(0.39, 7.34) | 2.42(-1.32, 6.16) | 10.5(2.32, 18.68) |
| **p-value** | <.0001 | <.0001 | <.0001 | 0.03 | 0.1993 | 0.1471 |
| **β (95%CI)**** | 2.29(1.64, 2.95) | 2.4(1.65, 3.15) | 0.03(-1.76, 1.82) | 2.19(1.4, 2.98) | 2.23(1.33, 3.12) | 0.01(-1.96, 1.98) |
| **p-value** | <.0001 | <.0001 | 0.9733 | <.0001 | <.0001 | 0.9931 |
| **FHA** | | | | | | |
| **α (95% CI)*** | 3.3(2.91, 3.69) | 3.3(2.91, 3.69) | 3.22(2.68, 3.75) | 7.42(5.01, 9.83) | 6.77(3.76, 9.78) | 3.96(0.33, 7.58) |
| **p-value** | <.0001 | 0.3005 | <.0001 | <.0001 | 0.7955 | 0.033 |
| **β (95%CI)**** | 2.15(1.6, 2.71) | 2.12(1.43, 2.81) | 0.44(-0.36, 1.24) | 1.87(1.32, 2.41) | 2.11(1.39, 2.82) | -0.06(-0.91, 0.8) |
| **p-value** | <.0001 | <.0001 | 0.3182 | <.0001 | <.0001 | 0.8948 |
| **PRN** | | | | | | |
| **α (95% CI)*** | 0.02(-0.54, 0.58) | 0.02(-0.54, 0.58) | 3.58(2.73, 4.43) | 3.36(-0.75, 7.47) | 1.56(-2.51, 5.64) | 4.39(-1.59, 10.38) |
| **p-value** | 0.9423 | 0.9423 | <.0001 | 0.1074 | 0.4455 | 0.1471 |
| **β (95%CI)**** | 2.65(1.84, 3.46) | 2.66(1.91, 3.42) | 0.1(-1.14, 1.35) | 2.34(1.42, 3.25) | 2.58(1.63, 3.53) | -0.32(-1.71, 1.07) |
| **p-value** | <.0001 | <.0001 | 0.8694 | <.0001 | <.0001 | 0.6464 |
| **FIM** | | | | | | |
| **α (95% CI)*** | 0.36(-0.33, 1.06) | 0.36(-0.33, 1.06) | 4.67(3.65, 5.68) | 2.41(-3.07, 7.88) | 0.6(-3.99, 5.18) | 6.34(-1.41, 14.09) |
| **p-value** | 0.3005 | 0.3005 | <.0001 | 0.3828 | 0.7955 | 0.107 |
| **β (95%CI)**** | 3.23(2.27, 4.19) | 3.04(2.21, 3.86) | -0.74(-2.2, 0.72) | 3.16(1.92, 4.4) | 2.58(1.51, 3.65) | -0.19(-2, 1.63) |
| **p-value** | <.0001 | <.0001 | 0.3182 | <.0001 | <.0001 | 0.8377 |

*Note: Linear regression models using the log2 transformed antibody titers were used to determine the impact of maternal titers at the time of delivery on infant vaccine response at specified time points.*α is the regression intercept. *** *Negative β coefficients imply that infant antibody titers decline with increasing maternal antibody levels at delivery, positive numbers imply that infant antibody titers increase with increasing maternal antibody levels at delivery and a β coefficient of zero indicates no association between infant antibody titers and maternal antibody levels at delivery*. **** P-value<0.05 was considered significant. Abbreviations: FHA= Filamentous hemagglutinin, FIM= Fimbriae type 2/3, PRN= Pertactin, and PTx= Pertussis toxin.*

# **Table S3**. Association between maternal antibody titers (at the time of delivery) and infant antibody titers as measured by linear regression coefficient, β, with 95% CIs - for children who received three doses of DTwP vaccine (n=28), per pertussis antigen at various time points in both unadjusted and adjusted (for birth weight, gestational age and gender) models.

| ***Regression Coefficients (α,β) with 95% confidence interval & P-values****** | **Unadjusted Model** | | | **Adjusted Model** | | |
| --- | --- | --- | --- | --- | --- | --- |
|  | **At Birth** | **At 6 weeks** | **At 18 weeks** | **At Birth** | **At 6 weeks** | **At 18 weeks** |
| **PTx** | | | | | | |
| **α (95% CI)*** | 0.46(-0.72, 1.65) | -0.93(-2.25, 0.4) | 3.4(0.58, 6.23) | 4.76(-8.08, 17.6) | 5.06(-7.97, 18.09) | 20.38(1.35, 39.41) |
| **p-value** | 0.4281 | 0.1592 | 0.0205 | 0.4414 | 0.4139 | 0.0376 |
| **β (95%CI)**** | 0.73(0.33, 1.13) | 0.7(0.23, 1.17) | 0.15(-0.84, 1.15) | 0.68(0.02, 1.34) | 0.59(-0.16, 1.33) | -0.02(-1.06, 1.02) |
| **p-value** | 0.001 | 0.0055 | 0.7513 | 0.0445 | 0.1118 | 0.9685 |
| **FHA** | | | | | | |
| **α (95% CI)*** | 1.25(0.24, 2.27) | 0.37(-1.05, 1.78) | 4.07(1.9, 6.25) | 4.51(-0.57, 9.59) | 6.87(-1.41, 15.15) | 9.96(-0.37, 20.28) |
| **p-value** | 0.0173 | 0.5955 | 0.0008 | 0.078 | 0.0956 | 0.0576 |
| **β (95%CI)**** | 0.78(0.57, 0.99) | 0.67(0.38, 0.96) | 0.02(-0.43, 0.48) | 0.62(0.34, 0.91) | 0.44(-0.06, 0.94) | 0.01(-0.6, 0.63) |
| **p-value** | <.0001 | 0.0001 | 0.919 | 0.0003 | 0.0773 | 0.9671 |
| **PRN** | | | | | | |
| **α (95% CI)*** | -0.74(-1.7, 0.22) | -1.42(-2.22, -0.62) | 4.56(2.97, 6.16) | 3.14(-4.49, 10.77) | -0.95(-7.86, 5.96) | 10.01(-2.04, 22.06) |
| **p-value** | 0.1244 | 0.0014 | <.0001 | 0.3941 | 0.7699 | 0.0964 |
| **β (95%CI)**** | 0.92(0.64, 1.2) | 0.74(0.49, 1) | 0.18(-0.32, 0.69) | 0.7(0.31, 1.09) | 0.72(0.34, 1.1) | 0.18(-0.52, 0.87) |
| **p-value** | <.0001 | <.0001 | 0.4535 | 0.0017 | 0.0015 | 0.595 |
| **FIM** | | | | | | |
| **α (95% CI)*** | -0.02(-1.09, 1.05) | -0.09(-1.06, 0.89) | 5.83(3.99, 7.67) | -2.86(-14.72, 9) | -3.03(-12.99, 6.92) | 23.85(6.95, 40.74) |
| **p-value** | 0.9704 | 0.8522 | <.0001 | 0.6151 | 0.5194 | 0.009 |
| **β (95%CI)**** | 0.83(0.49, 1.18) | 0.62(0.26, 0.97) | 0.11(-0.6, 0.82) | 0.77(0.32, 1.23) | 0.56(0.14, 0.97) | 0.08(-0.63, 0.8) |
| **p-value** | <.0001 | 0.0017 | 0.7518 | 0.0026 | 0.0127 | 0.8074 |

*Note: Linear regression models using the log2 transformed antibody titers were used to determine the impact of maternal titers at the time of delivery on infant vaccine response at specified time points.*α is the regression intercept. *** *Negative β coefficients imply that infant antibody titers decline with increasing maternal antibody levels at delivery, positive numbers imply that infant antibody titers increase with increasing maternal antibody levels at delivery and a β coefficient of zero indicates no association between infant antibody titers and maternal antibody levels at delivery*. **** P-value<0.05 was considered significant. Abbreviations: FHA= Filamentous hemagglutinin, FIM= Fimbriae type 2/3, PRN= Pertactin, and PTx= Pertussis toxin.*

**Table S4.** Influence of maternal antibody titers (at the time of delivery) - which were dichotomized as above or below median (relative to below median category of maternal antibody titers)-on infant vaccine response, for children who received three doses of DTwP vaccine (n=28), per pertussis antigen at various time points in both unadjusted and adjusted (for birth weight, gestational age and gender) models.

| ***Regression Coefficients (α,β) with 95% confidence interval & P-values****** | **Unadjusted Model** | | | **Adjusted Model** | | |
| --- | --- | --- | --- | --- | --- | --- |
|  | **At Birth** | **At 6 weeks** | **At 18 weeks** | **At Birth** | **At 6 weeks** | **At 18 weeks** |
| **PTx** | | | | | | |
| **α (95% CI)*** | 0.97(-0.06, 2) | -0.14(-1.23, 0.95) | 3.2(1.03, 5.37) | 3.87(0.39, 7.34) | 2.42(-1.32, 6.16) | 10.5(2.32, 18.68) |
| **p-value** | 0.0646 | 0.7914 | 0.0057 | 0.03 | 0.1993 | 0.0128 |
| **β (95%CI)**** | 2.51(1.02, 4) | 1.97(0.27, 3.68) | 1.32(-2.04, 4.68) | 2.19(1.4, 2.98) | 2.23(1.33, 3.12) | 0.01(-1.96, 1.98) |
| **p-value** | 0.002 | 0.0256 | 0.4235 | <.0001 | <.0001 | 0.9931 |
| **FHA** | | | | | | |
| **α (95% CI)*** | 3.28(2.76, 3.79) | 2.33(1.48, 3.18) | 4.45(3.41, 5.49) | 7.42(5.01, 9.83) | 6.77(3.76, 9.78) | 3.96(0.33, 7.58) |
| **p-value** | <.0001 | <.0001 | <.0001 | <.0001 | <.0001 | 0.033 |
| **β (95%CI)**** | 2.5(1.84, 3.16) | 1.8(0.73, 2.86) | -0.48(-1.84, 0.89) | 1.87(1.32, 2.41) | 2.11(1.39, 2.82) | -0.06(-0.91, 0.8) |
| **p-value** | <.0001 | 0.0013 | 0.9183 | <.0001 | <.0001 | 0.8948 |
| **PRN** | | | | | | |
| **α (95% CI)*** | 0.09(-0.82, 0.99) | -0.75(-1.5, -0.01) | 4.64(3.34, 5.95) | 3.36(-0.75, 7.47) | 1.56(-2.51, 5.64) | 4.39(-1.59, 10.38) |
| **p-value** | 0.848 | 0.0482 | <.0001 | 0.1074 | 0.4455 | 0.1471 |
| **β (95%CI)**** | 3.38(2.13, 4.64) | 2.71(1.55, 3.88) | 0.84(-1.09, 2.77) | 2.34(1.42, 3.25) | 2.58(1.63, 3.53) | -0.32(-1.71, 1.07) |
| **p-value** | <.0001 | <.0001 | 0.3741 | <.0001 | <.0001 | 0.6464 |
| **FIM** | | | | | | |
| **α (95% CI)*** | 0.18(-1.31, 1.67) | -0.25(-1.26, 0.76) | 6.03(4.03, 8.04) | 2.41(-3.07, 7.88) | 0.6(-3.99, 5.18) | 6.34(-1.41, 14.09) |
| **p-value** | 0.8059 | 0.6108 | <.0001 | 0.3828 | 0.7955 | 0.107 |
| **β (95%CI)**** | 2.87(0.63, 5.12) | 3(1.33, 4.66) | -0.16(-3.44, 3.11) | 3.16(1.92, 4.4) | 2.58(1.51, 3.65) | -0.19(-2, 1.63) |
| **p-value** | 0.0144 | 0.0013 | 0.9183 | <.0001 | <.0001 | 0.8377 |

*Note: Linear regression models using the log2 transformed antibody titers were used to determine the impact of maternal titers at the time of delivery on infant vaccine response at specified time points.*α is the regression intercept. *** *Negative β coefficients imply that infant antibody titers decline with increasing maternal antibody levels at delivery, positive numbers imply that infant antibody titers increase with increasing maternal antibody levels at delivery and a β coefficient of zero indicates no association between infant antibody titers and maternal antibody levels at delivery*. **** P-value<0.05 was considered significant. Abbreviations: FHA= Filamentous hemagglutinin, FIM= Fimbriae type 2/3, PRN= Pertactin, and PTx= Pertussis toxin.*

# **Table S5.** Association between maternal antibody titers (at the time of delivery) and infant antibody titers as measured by linear regression coefficient, β, with 95% CIs for children who received at least two doses (n=129) of DTwP vaccine, per pertussis antigen at various time points in both unadjusted and adjusted (adjusted for birth weight, gestational age, gender and breast feeding status) linear regression models.

| ***Regression Coefficients (α,β) with 95% confidence interval & P-values****** | **Unadjusted Model** | | | **Adjusted Model** | | |
| --- | --- | --- | --- | --- | --- | --- |
|  | **At Birth** | **At 6 weeks** | **At 18 weeks** | **At Birth** | **At 6 weeks** | **At 18 weeks** |
| **PTx** | | | | | | |
| **α (95% CI)*** | 0.12(-0.32, 0.57) | -1.43(-1.96, -0.9) | 2.75(1.38, 4.13) | 3(-0.19, 6.19) | 1.29(-2.41, 4.98) | 9.95(1.2, 18.7) |
| **p-value** | 0.5834 | <.0001 | 0.0001 | 0.0645 | 0.487 | 0.0265 |
| **β (95%CI)**** | 0.83(0.67, 0.99) | 0.9(0.71, 1.09) | 0.14(-0.37, 0.64) | 0.77(0.59, 0.96) | 0.79(0.55, 1.03) | 0.07(-0.49, 0.63) |
| **p-value** | <.0001 | <.0001 | 0.5863 | <.0001 | <.0001 | 0.8048 |
| **FHA** | | | | | | |
| **α (95% CI)*** | 0.68(0.12, 1.24) | -0.33(-1.1, 0.45) | 2.86(1.82, 3.89) | 3.69(1.52, 5.85) | 2.61(-0.77, 6) | 4.36(0.22, 8.51) |
| **p-value** | 0.0172 | 0.4064 | <.0001 | 0.0012 | 0.1271 | 0.0393 |
| **β (95%CI)**** | 0.86(0.74, 0.99) | 0.83(0.66, 1) | 0.14(-0.1, 0.37) | 0.75(0.61, 0.88) | 0.73(0.52, 0.95) | 0.02(-0.26, 0.29) |
| **p-value** | <.0001 | <.0001 | 0.7987 | <.0001 | <.0001 | 0.9059 |
| **PRN** | | | | | | |
| **α (95% CI)*** | -1.22(-1.79, -0.66) | -1.61(-2.2, -1.02) | 3.08(2.07, 4.08) | 1.79(-2.02, 5.6) | 0.39(-3.91, 4.68) | 5.08(-1.22, 11.38) |
| **p-value** | <.0001 | <.0001 | <.0001 | 0.3525 | 0.8572 | 0.1121 |
| **β (95%CI)**** | 1.03(0.84, 1.23) | 0.83(0.63, 1.03) | 0.23(-0.11, 0.57) | 0.91(0.68, 1.14) | 0.79(0.53, 1.05) | 0.02(-0.39, 0.42) |
| **p-value** | <.0001 | <.0001 | 0.1757 | <.0001 | <.0001 | 0.9276 |
| **FIM** | | | | | | |
| **α (95% CI)*** | 0.12(-0.4, 0.64) | -0.45(-0.93, 0.03) | 4.23(3.28, 5.19) | -0.1(-4.26, 4.06) | -0.1(-4.26, 4.06) | 6.43(-1.56, 14.43) |
| **p-value** | 0.6446 | 0.0659 | <.0001 | 0.9627 | 0.9627 | 0.1126 |
| **β (95%CI)**** | 0.88(0.72, 1.04) | 0.74(0.59, 0.89) | 0.04(-0.27, 0.35) | 0.65(0.46, 0.84) | 0.65(0.46, 0.84) | 0.18(-0.2, 0.57) |
| **p-value** | <.0001 | <.0001 | 0.7987 | <.0001 | <.0001 | 0.3415 |

*Note: Linear regression models using the log2 transformed antibody titers were used to determine the impact of maternal titers at the time of delivery on infant vaccine response at specified time points.*α is the regression intercept. *** *Negative β coefficients imply that infant antibody titers decline with increasing maternal antibody levels at delivery, positive numbers imply that infant antibody titers increase with increasing maternal antibody levels at delivery and a β coefficient of zero indicates no association between infant antibody titers and maternal antibody levels at delivery*. **** P-value<0.05 was considered significant. Abbreviations: FHA= Filamentous hemagglutinin, FIM= Fimbriae type 2/3, PRN= Pertactin, and PTx= Pertussis toxin.*

# **Table S6.** Influence of maternal antibody titers- which were dichotomized as above or below median (relative to below median category of maternal antibody titers) - on infant vaccine response, for children who received at least two doses (n=129) of DTwP vaccine, per pertussis antigen at various time points in both unadjusted and adjusted (for birth weight, gestational age, gender and breast feeding status) models.

| ***Regression Coefficients (α,β) with 95% confidence interval & P-values****** | **Unadjusted Model** | | | **Adjusted Model** | | |
| --- | --- | --- | --- | --- | --- | --- |
|  | **At Birth** | **At 6 weeks** | **At 18 weeks** | **At Birth** | **At 6 weeks** | **At 18 weeks** |
| **PTx** | | | | | | |
| **α (95% CI)*** | 0.97(-0.06, 2) | -0.14(-1.23, 0.95) | 3.2(1.03, 5.37) | 6.39(-8.26, 21.05) | 7.6(-6, 21.2) | 19.29(-0.04, 38.62) |
| **p-value** | 0.0646 | 0.7914 | 0.0057 | 0.3652 | 0.2444 | 0.0505 |
| **β (95%CI)**** | 2.51(1.02, 4) | 1.97(0.27, 3.68) | 1.32(-2.04, 4.68) | 1.85(-0.88, 4.57) | 1.25(-1.47, 3.98) | 0.34(-3.44, 4.11) |
| **p-value** | 0.002 | 0.0256 | 0.4235 | 0.1679 | 0.3335 | 0.8499 |
| **FHA** | | | | | | |
| **α (95% CI)*** | 3.28(2.76, 3.79) | 2.33(1.48, 3.18) | 4.45(3.41, 5.49) | 7.11(3.58, 10.65) | 8.37(2.86, 13.89) | 9.81(1.69, 17.93) |
| **p-value** | <.0001 | <.0001 | <.0001 | 0.0007 | 0.0066 | 0.0216 |
| **β (95%CI)**** | 2.5(1.84, 3.16) | 1.8(0.73, 2.86) | -0.48(-1.84, 0.89) | 2.01(1.3, 2.72) | 1.84(0.61, 3.08) | 0.09(-1.7, 1.88) |
| **p-value** | <.0001 | 0.0013 | 0.9183 | <.0001 | 0.0072 | 0.9175 |
| **PRN** | | | | | | |
| **α (95% CI)*** | 0.09(-0.82, 0.99) | -0.75(-1.5, -0.01) | 4.64(3.34, 5.95) | 0.65(-7.04, 8.34) | -5.96(-11.41, -0.51) | 8.26(-5.27, 21.79) |
| **p-value** | 0.848 | 0.0482 | <.0001 | 0.8589 | 0.0348 | 0.2101 |
| **β (95%CI)**** | 3.38(2.13, 4.64) | 2.71(1.55, 3.88) | 0.84(-1.09, 2.77) | 3.19(1.65, 4.73) | 3.68(2.54, 4.83) | 1.14(-1.78, 4.06) |
| **p-value** | <.0001 | <.0001 | 0.3741 | 0.0006 | <.0001 | 0.4131 |
| **FIM** | | | | | | |
| **α (95% CI)*** | 0.18(-1.31, 1.67) | -0.25(-1.26, 0.76) | 6.03(4.03, 8.04) | -2.81(-17.92, 12.3) | -4.79(-16.25, 6.68) | 23.96(6.25, 41.66) |
| **p-value** | 0.8059 | 0.6108 | <.0001 | 0.6964 | 0.3781 | 0.0119 |
| **β (95%CI)**** | 2.87(0.63, 5.12) | 3(1.33, 4.66) | -0.16(-3.44, 3.11) | 2.8(-0.26, 5.85) | 2.69(0.25, 5.12) | 0.28(-3.43, 3.98) |
| **p-value** | 0.0144 | 0.0013 | 0.9183 | 0.0698 | 0.0335 | 0.8751 |

*Note: Linear regression models using the log2 transformed antibody titers were used to determine the impact of maternal titers at the time of delivery on infant vaccine response at specified time points.*α is the regression intercept. *** *Negative β coefficients imply that infant antibody titers decline with increasing maternal antibody levels at delivery, positive numbers imply that infant antibody titers increase with increasing maternal antibody levels at delivery and a β coefficient of zero indicates no association between infant antibody titers and maternal antibody levels at delivery*. **** P-value<0.05 was considered significant. Abbreviations: FHA= Filamentous hemagglutinin, FIM= Fimbriae type 2/3, PRN= Pertactin, and PTx= Pertussis toxin.*

# **Table S7.** Comparison of maternal demographics among those who received 1 vs 2 vs 3 doses of DTwP.

| **Maternal Characteristics** | **DTwP 1 dose** | **DTwP 2 doses** | **DTwP 3 doses** | **Overall** | **p-value** |
| --- | --- | --- | --- | --- | --- |
|  | **N (%)** | **N (%)** | **N (%)** | **N (%)** |  |
| **Maternal age** | | | | | |
| 16-20 years | 11 (20.8) | 11 (16.7) | 5 (19.2) | 27 (18.6) | 0.388 |
| 21-25 years | 12 (22.6) | 19 (28.8) | 9 (34.6) | 40 (27.6) |  |
| 26-30 years | 20 (37.7) | 25 (37.9) | 4 (15.4) | 49 (33.8) |  |
| >30 years | 10 (18.9) | 11 (16.7) | 8 (30.8) | 29 (20) |  |
| Mean±SD | 26.5±6.2 | 26.6±5.1 | 26.7±6.6 | 26.5±5.7 |  |
| n | 53 | 66 | 26 | 145 |  |
| **Maternal education** | | | | | |
| No Formal Education | 36 (67.9) | 43 (65.2) | 12 (46.2) | 91 (62.8) | 0.147 |
| Formal Education Received | 17 (32.1) | 23 (34.9) | 14 (53.9) | 54 (37.2) |  |
| n | 53 | 66 | 26 | 145 |  |
| **ANC** | | | | | |
| None | 27 (50.9) | 30 (45.5) | 12 (46.2) | 69 (47.6) | 0.435 |
| 1 | 1 (1.9) | 4 (6.1) | 0 (0) | 5 (3.5) |  |
| 2 | 2 (3.8) | 5 (7.6) | 4 (15.4) | 11 (7.6) |  |
| 3 | 4 (7.6) | 8 (12.1) | 1 (3.9) | 13 (9) |  |
| >3 | 19 (35.9) | 19 (28.8) | 9 (34.6) | 47 (32.4) |  |
| n | 53 | 66 | 26 | 145 |  |
| **ANC skilled provider** | | | | | |
| Any skilled provider | 20 (95.2) | 22 (78.6) | 7 (77.8) | 49 (84.5) | 0.234 |
| Others | 1 (4.8) | 6 (21.4) | 2 (22.2) | 9 (15.5) |  |
| n | 21 | 28 | 9 | 58 |  |
| **ANC skilled provider** | | | | | |
| None | 21 (39.6) | 28 (42.4) | 10 (38.5) | 59 (40.7) | 0.846 |
| 1 | 16 (30.2) | 20 (30.3) | 6 (23.1) | 42 (29) |  |
| 2 | 16 (30.2) | 17 (25.8) | 10 (38.5) | 43 (29.7) |  |
| 3 | 0 (0) | 1 (1.5) | 0 (0) | 1 (0.7) |  |
| n | 53 | 66 | 26 | 145 |  |
| **Body Mass Index** | | | | | |
| Underweight | 1 (2.8) | 0 (0) | 0 (0) | 1 (1.2) | 0.722 |
| Normal | 14 (38.9) | 14 (37.8) | 7 (50) | 35 (40.2) |  |
| Overweight/Obese | 21 (58.3) | 23 (62.2) | 7 (50) | 51 (58.6) |  |
| n | 36 | 37 | 14 | 87 |  |
| **Hypertension** | | | | | |
| Yes | 0 (0) | 1 (2.5) | 1 (6.3) | 2 (2.1) | 0.34 |
| No | 38 (100) | 39 (97.5) | 15 (93.8) | 92 (97.9) |  |
| n | 38 | 40 | 16 | 94 |  |
| **TT vaccines doses** | | | | | |
| None | 21 (39.6) | 28 (42.4) | 10 (38.5) | 59 (40.7) | 0.846 |
| 1 | 16 (30.2) | 20 (30.3) | 6 (23.1) | 42 (29) |  |
| 2 | 16 (30.2) | 17 (25.8) | 10 (38.5) | 43 (29.7) |  |
| 3 | 0 (0) | 1 (1.5) | 0 (0) | 1 (0.7) |  |
| n | 53 | 66 | 26 | 145 |  |

*Note: Maternal education was assessed in terms of the number of completed years of schooling. Chi-square test was used to compare maternal demographics among mothers of infants who received 1 vs 2 vs* 3 *doses of DTwP. P-value<0.05 was considered significant. Abbreviations: DTwP=* *Diphtheria, Tetanus* *and whole cell Pertussis vaccination, ANC= Antenatal care, TT= Tetanus Toxoid.*

# **Table S8.** Comparison of infant demographics among those who received 1 vs 2 vs 3 doses of DTwP.

| **Infant characteristics** | **DTwP 1 dose** | **DTwP 2 doses** | **DTwP 3 doses** | **Overall** | **p-value** |
| --- | --- | --- | --- | --- | --- |
|  | **N (%)** | **N (%)** | **N (%)** | **N (%)** |  |
| **Gender** | | | | | |
| Male | 41 (65.1) | 37 (50.7) | 13 (46.4) | 91 (55.5) | 0.138 |
| Female | 22 (34.9) | 36 (49.3) | 15 (53.6) | 73 (44.5) |  |
| n | 63 | 73 | 28 | 164 |  |
| **Age at first blood draw(days)** | | | | | |
| Mean±SD | 2.1±2.4 | 2±2.4 | 2.5±2.8 | 2.1±2.5 | 0.586 |
| n | 63 | 73 | 28 | 164 |  |
| **Weight(Kg)** | | | | | |
| Mean±SD | 2.75±0.38 | 2.79±0.44 | 2.79±0.35 | 2.78±0.4 | 0.834 |
| n | 61 | 72 | 27 | 160 |  |
| **Length (cm)** | | | | | |
| Mean±SD | 48.8±5.6 | 47.9±2.4 | 47.6±2.5 | 48.2±3.9 | 0.314 |
| n | 54 | 70 | 27 | 151 |  |
| **Head Circumference(cm)** | | | | | |
| Mean±SD | 33.3±1.5 | 33.3±1.6 | 33.4±1.1 | 33.3±1.5 | 0.932 |
| n | 54 | 70 | 27 | 151 |  |
| **Gestational age at birth (weeks)** | | | | | |
| <37 Weeks | 33 (62.3) | 36 (62.1) | 12 (54.6) | 81 (60.9) | 0.799 |
| >=37 Weeks | 20 (37.7) | 22 (37.9) | 10 (45.5) | 52 (39.1) |  |
| Mean±SD | 34.8±5.9 | 35.5±3.6 | 36.3±3.8 | 35.4±4.7 |  |
| n | 53 | 58 | 22 | 133 |  |

*Note: Infants were vaccinated according to the EPI 6-10-14 week immunization schedule. One-way ANOVA was used to compare means among infants who received 1 vs 2 vs* 3 *doses of DTwP. P-value<0.05 was considered significant.*

# **Table S9.** Comparison between Ab titers in cord blood vs those obtained within 72 hours of delivery.

| **Antibody titers at birth** | **Cord-Blood** | | **Peripheral** | | **p-value** |
| --- | --- | --- | --- | --- | --- |
|  | **Mean** | **95% CI** | **Mean** | **95% CI** |  |
| **n** | 63 | | 30 | |  |
| **PTx** | 7.5 | 5.5-9.4 | 13.4 | 3.0-23.8 | 0.259 |
| **FHA** | 40.6 | 25.3-55.9 | 32.1 | 18.2-46.0 | 0.408 |
| **PRN** | 13.1 | -0.4-26.5 | 6.6 | 4.0-9.1 | 0.346 |
| **FIM** | 22.6 | 11.6-33.5 | 15.5 | 0.8-30.2 | 0.439 |

*Note: T-Test with un-equal variances was used to compare the two* groups. *Peripheral blood was collected within 72 hours of delivery via a venipuncture. P-value<0.05 was considered significant. Abbreviations: FHA= Filamentous hemagglutinin, FIM= Fimbriae type 2/3, PRN= Pertactin, and PTx= Pertussis toxin.*

# **Table S10.** Variability in timing of DTwP doses

| **Timing of dose** | **Median (IQR)** |
| --- | --- |
| Age in weeks of dose1 | 6.9 (6.1,9.5) |
| Age in weeks of dose2 | 12.1 (10.9,14.1) |
| Age in weeks of dose3 | 15.3 (14.9,15.7) |

*Note: Infants were vaccinated according to the*

*EPI 6-10-14 week immunization schedule.*

# **Fig S1**. Maternal and infant pertussis antibody titers in those who were unvaccinated compared to those who received at least two doses of DTwP vaccine (n=129). Pregnancy = Maternal sera; Birth = Infant cord blood/sera; 6 and 18 weeks = Infant sera

# **Fig S2**. Geometric mean concentrations (GMC) with 95% confidence interval (CI) of infant pertussis antibody titers and their half-lives for infants who did not receive any vaccination. *Abbreviations*: *FHA= Filamentous hemagglutinin, FIM= Fimbriae type 2/3, PRN= Pertactin, and PTx= Pertussis toxin*. *Note: I bars indicate 95% CIs.*(------- one half infant antibody titer at birth). Outliers (defined as greater than two standard deviations from the mean) were included (See Figure 2 for GMC with 95% confidence intervals with outliers excluded).

**Fig S3**. Consort Diagram

# **Fig S4.** Comparison of Geometric mean concentrations (GMC) of infant antibody titers who received at least two doses of DTwP vaccine-per pertussis antigen at various time points- in those with high maternal antibody titers at delivery (right) compared to those with low maternal antibody titers at delivery (left). Maternal antibody titers at delivery were dichotomized as above or below median.
